# Why Pragmatics and Theory of Mind Do Not (Completely) Overlap

**DOI:** 10.3389/fpsyg.2018.01453

**Published:** 2018-08-13

**Authors:** Francesca M. Bosco, Maurizio Tirassa, Ilaria Gabbatore

**Affiliations:** ^1^Department of Psychology, University of Turin, Turin, Italy; ^2^Neuroscience Institute of Turin (NIT), University of Turin, Turin, Italy; ^3^Faculty of Humanities, University of Oulu, Oulu, Finland

**Keywords:** pragmatics, theory of mind, experimental pragmatics, inferential ability, cognitive pragmatics

## Abstract

Aim of the paper is to discuss the extent to which pragmatics, i.e., the ability to use language and other expressive means to convey meaning in a specific interactional context, overlaps with Theory of Mind (ToM), i.e., the ability to ascribe mental states to oneself and the others. We present empirical data available in the current literature concerning the relation between these two faculties, with specific reference to the developmental and clinical domains. Part of the literature we take into account appears to show that ToM does correlate with pragmatic ability; however, other studies appear to show that pragmatic ability alone cannot explain the empirical differences of performance across different kinds of pragmatic tasks, and therefore that another, at least partially different faculty is required to account for human communication. We argue that to conceive pragmatics as a sort of subcomponent of ToM, and thus to conflate or reduce the notion of pragmatics into the (wider) notion of ToM, is not theoretically correct and a possible cause of methodological confusion in the relevant empirical research. It thus turns out to be necessary that the two faculties be investigated with separate theories as well as different experimental tasks.

## Introduction

Pragmatics is a complex theoretical construct for which several definitions have been proposed. Among others, these include the study of the speaker’s meaning in relation to the use of language, the relationship between signs and their users, the ability to use language and other expressive means (like gestures, body movements, facial expressions, and paralinguistic cues) to convey communicative meanings in a context, the ability to manage conversations, discourse analysis (see Levinson, 1983; Tirassa, 1999; Mey, 2001; Cummings, 2005; Huang, 2007; Tirassa and Bosco, 2008; Bara, 2010).

Behind such an array of perspectives, there is unanimous agreement that pragmatic ability requires a partner more than merely comprehend the literal meaning of an utterance, and that this involves some kind of inferential processes to fill the gap between what a speaker literally has said and what she actually meant (Austin, 1962; Searle, 1979; Grice, 1989). The ability to infer the communicative intention behind an utterance thus is what characterizes human communication. This is also necessary to distinguish between different possible interpretations of the same literal act. For example a person might say “What a wonderful day!” for (at least) two reasons: sincerely, if the sky is blue and the sun is shining, or ironically, if it is cold and rainy. Thus, the same statement can be sincere, ironic, etc., depending on the context in which it is uttered (Bosco et al., 2004; Bosco and Bucciarelli, 2008). Other cases where the literal meaning does not correspond to the intended meaning are indirect speech acts, e.g., “Can you pass the salt,” or figurative language, like metaphors (e.g., “Lawyers are sharks”), idioms (e.g., “To walk in someone else’s shoes”), hyperboles (e.g., “She’s a genius,” referring to a brilliant person), and so on.

Theory of Mind (ToM) appears comparatively simpler to define, at least at first sight. This label was originally coined by Premack and Woodruff (1978) to refer to the ability to attribute mental states to oneself and to the others and to use such attribution to predict and explain behavior. Since then, however, this originally “single” ability has been decomposed into several facets or subcomponents, such as first-person and third-person ToM (i.e., respectively, the ability to understand one’s own beliefs and those of another person: Nichols and Stich, 2003), affective ToM and cognitive ToM (Tager-Flusberg and Sullivan, 2000), first-order and second-order ToM (i.e., respectively, the ability to understand someone’s beliefs about a state of the world and the more complex ability to understand someone’s beliefs about someone else’s belief: Wimmer and Perner, 1983). We refer the interested reader to Dimopoulou et al. (2017) for a recent review of this research area and to Brizio et al. (2015) for ToM development.

Sperber and Wilson (2002) proposed to view pragmatics as a sub-module of mind-reading, the latter being in practice a synonym of ToM. Based on this, several studies have used pragmatic tasks, defining them as ToM tasks. A clear example is the Strange Stories test (Happé, 1994), one of the most used ways to investigate advanced ToM. The test consists of 24 story-items (two for each type) concerning Pretense, Joke, Lie, White Lie, Appearance/Reality, Double Bluff, Contrary Emotions, Forgetting, Misunderstanding, Persuasion, Figure of Speech and Irony; in the field of pragmatics the last four tasks - Misunderstanding, Persuasion, Figure of Speech and Irony - would be considered examples of pragmatic tasks. A token example of Strange Story is: *Ann’s mother has spent a long time cooking Ann’s favorite meal, fish and chips. But when she brings it to Ann, she is watching TV, and she doesn’t even look up, or say thank you. Ann’s mother is cross and says “Well, that’s very nice, isn’t it! That is what I call politeness!”* The subject is asked “Is it true what Ann’s mother says?” and “Why does Ann’s mother say this?” The item is considered passed if both questions are answered correctly.

In our view this story involves both pragmatic competence, dealing with language and its use in the given context, and TOM competence, dealing with the mental states that may help explain such use of language. A correlation between a purely pragmatic task and the Strange Stories could then be explained either with a purely pragmatic faculty (if the second question is conceived of as focusing on language use) or an overlap or synergy of pragmatic and TOM abilities (if the second question is viewed as a TOM question independent of language use). Under no respect should it count as evidence that pragmatics straightforwardly identifies with TOM. It could be no coincidence that validating Strange Stories on a sample of healthy children, has yielded a Cronbach’s alpha coefficient (α) = 0.65 (Hayward and Homer, 2017). This coefficient (Cronbach, 1951) is a reliability measure of the intercorrelation among the items composing a test; according to Devellis (2012), an α between 0.60 and 0.70 should be considered undesirable or minimally acceptable.

Substantially the same argument can be done for other, similar tests like, for example, those developed by Champagne-Lavau et al. (2012) and Champagne-Lavau and Charest (2015), who used open questions about a speaker’s ironic intent as an assessment of ToM in patients with schizophrenia.

What we want to argue is that to conflate or reduce the notion of pragmatics into the (wider) notion of ToM is theoretically unsound and may cause methodological confusion in the relevant empirical research. Of course there are relationships between the two faculties but they nonetheless remain such: two distinct faculties of the human mind whose roles, domains of intervention, and ways of functioning overlap but are not identical. That ToM plays a role in communication can hardly be denied. Nevertheless, recent studies showed that ToM alone cannot explain the performance of children (Bosco and Gabbatore, 2017a,b) and of persons with schizophrenia (Bosco et al., 2012) at a variety of pragmatic tasks. Furthermore, the impairments of pragmatics and ToM in schizophrenia do not overlap completely (Bambini et al., 2016; Parola et al., 2018). As will be discussed in the next session, to use the comprehension of pragmatic tasks, including irony or the comprehension of figurative language, as a straightforward measure of ToM raises problems of content validity: therefore, different tasks should be used in the empirical practice to investigate the two constructs.

## Empirical Studies Of The Relationship Between Pragmatics And Theory Of Mind

### Studies of Children With Typical and Atypical Development

Several studies in the developmental domain (Ryder and Leinonen, 2003; Bosco et al., 2006, 2013; Loukusa et al., 2007; Glenwright and Pexman, 2010) found that pragmatic abilities improve with age, yielding, among the rest, a progressively better management of indirect speech acts (Bosco and Bucciarelli, 2008), deceits (Bussey, 1999), and irony (Filippova and Astington, 2008). Children begin to exploit language to lie at about 3 years of age (Lewis, 1993) and their ability to handle lies of increasing difficulty grows during the pre-school and school period (see Talwar and Crossman, 2011). Furthermore, they can typically recognize ironic utterances at around 6 years of age (Dews and Winner, 1997; Harris and Pexman, 2003) or sometimes even earlier (Loukusa and Leinonen, 2008; Angeleri and Airenti, 2014).

Like pragmatics, ToM also undergoes significant improvement during childhood (Wellman and Liu, 2004), with the acquisition of first-order (Wimmer and Perner, 1983) and, later, second-order abilities (Perner and Wimmer, 1985). Its development continues through adolescence (Dumontheil et al., 2010; Apperly, 2012; Bosco et al., 2014b, 2016).

As far as the relationship between ToM and pragmatics is concerned, several authors have stressed that the ability to understand someone’s mental states and their relation with behavior is an undisputed requirement of human communication (Happé and Loth, 2002; Sperber and Wilson, 2002; Tirassa et al., 2006a,b; Bosco et al., 2009; Cummings, 2015). For example, the ability to deceive in communication has been commonly explained based on the ability to understand and foresee the interlocutor’s mental states (Peskin, 1996; Polak and Harris, 1999; Lee, 2000; Ma et al., 2015). The same holds for irony (Sullivan et al., 1995). Nilsen et al. (2011), for example, found a correlation between the development of verbal irony and second-order ToM, whereby children around 8 years of age performed similarly to adults in acknowledging that listeners require contextual knowledge to comprehend irony.

Several studies (Bucciarelli et al., 2003; Bosco and Bucciarelli, 2008; Bosco et al., 2013, 2015) found that understanding irony is typically more difficult to children than deceits, explaining such difference in terms of the comparative complexity of the inferential processes involved. In both cases the contents of the utterance do not correspond to the speaker’s private knowledge; however, in irony, but not in deceit, they also contradict the knowledge which is shared between the interlocutors. Winner and Leekman (1991) proposed that the different degrees of difficulty experienced by children with the two kind of tasks may depend on deceit requiring first-order reasoning about beliefs, and irony requiring inferences about the speaker’s beliefs about the listener’s beliefs, i.e., second-order ToM (see also Hancock et al., 2000). Indeed, Sullivan et al. (1995) found that children start to distinguish lies from jokes at around the age of 7, following the acquisition of the capacity to attribute second-order mental states.

Still, the exact role of ToM in children’s pragmatic performance is not completely clear. In line with the literature discussed above, Bosco and Gabbatore (2017a) found a correlation between children’s performance at ToM tasks and their ability to handle ironies and deceits. Further investigation, though, showed a significant role of first-order ToM in explaining children’s performance at deceitful, but not at ironic communicative acts. Moreover, no effect of second-order ToM was found on children’s performance at any of the pragmatic tasks investigated (Bosco and Gabbatore, 2017a). ToM alone thus didn’t prove capable of explaining the pattern of performance in sincere (the easiest), deceitful, and ironic (the most difficult to handle) tasks. This is in line with the results of Angeleri and Airenti (2014) to the effect that, despite a significant association between ToM and irony comprehension, no direct effect of ToM on humor understanding was noticeable. Such evidence appear to be at odds with theories granting ToM (e.g., Happé, 1993), and specifically second-order ToM (e.g., Winner and Leekman, 1991), a central role in irony comprehension and, in general, in pragmatics. For a similar line of argumentation in explaining children’s ability to recognize and recover different kinds of communicative failures see also Bosco and Gabbatore (2017b).

The interplay of pragmatic abilities and ToM has relevant implications in Autism Spectrum Disorder (ASD): ASD children display a malfunctioning both of ToM (e.g., Baron-Cohen et al., 1985) and of several aspects of pragmatic competence (Rundblad and Annaz, 2010; Angeleri et al., 2016; see also Happé, 1993). The most recent diagnostic criteria for ASD (DSM-5; American Psychiatric Association [APA], 2013) include impairments in social communication and reciprocity as well as restricted interests and repetitive behaviors. ASD appears to be linked to a recently described condition called Social (pragmatic) Communication Disorder (SCD), characterized by difficulties in using language for social purposes, recognizing the social context of communicative interactions, understanding non-literal language (e.g., jokes, idioms, metaphors), and using non-verbal communicative behaviors. The two conditions share the difficulties in social communication, but there is no sign in SCD of the restricted interests and repetitive behaviors typical of ASD (see also Swineford et al., 2014). Actually, one of the main nosographic features of SCD is that it cannot be diagnosed in the presence of ASD, a dissociation that may be interpreted as an attempt to disentangle, instead of conflating, the relation between pragmatics and ToM in such clinical conditions.

### Clinical Adult Population

#### Schizophrenia

Deficits of both pragmatics and ToM constitute an integral part of schizophrenia (Frith, 1992). As far as pragmatics is concerned, several studies have found a worsened performance at the comprehension of communicative acts when their literal meaning does not correspond to the speakers’ intended meaning, as is the case of indirect speech acts (Corcoran et al., 1995; Corcoran, 2003), irony (Parola et al., 2018), and figurative language uses like metaphors and idioms (Langdon et al., 2002a; Tavano et al., 2008; Schettino et al., 2010). Persons with schizophrenia may also encounter difficulties in the comprehension of deceits (Frith and Corcoran, 1996) and of narratives and stories (Marini et al., 2008), in discourse production (Haas et al., 2015), in the recognition and recovery of failures in communication (Bosco et al., 2012), and in the recognition of violation of Grice’s maxims (Tényi et al., 2002; Mazza et al., 2008).

Frith (1992) was the first to propose that a deficit of ToM could explain the cognitive and behavioral abnormalities of schizophrenia. This hypothesis was widely confirmed (e.g., Corcoran et al., 1995, 1997; Frith and Corcoran, 1996; Sarfati and Hardy-Baylé, 1999; Mazza et al., 2001; Brüne, 2005; Bosco et al., 2009). Specifically, Frith (1992) proposed to explain the communicative-pragmatic deficit of schizophrenia as the consequence of a primary ToM deficit.

Following this suggestion, several studies found impairments of ToM co-occurring with impairments of pragmatics in a variety of tests of conversational and narrative ability (Abu-Akel, 1999; Champagne-Lavau et al., 2009), indirect speech acts and figurative language comprehension (Corcoran et al., 1995; Langdon et al., 2002a,b; Brüne and Bodenstein, 2005; Mo et al., 2008; Champagne-Lavau and Stip, 2010; Gavilán and García-Albea, 2011), and the recognition of the violation of Gricean maxims and other social norms of communication (Corcoran and Frith, 1996; Mazza et al., 2008).

However, in several such studies ToM was assessed precisely with pragmatic tasks like indirect speech acts (Corcoran et al., 1995), irony (Mitchley et al., 1998), or the appreciation of the adherence to or violation of Gricean maxims (Corcoran and Frith, 1996). Of course, the real extent of the correlation between any two abilities (or impairments thereof) can hardly be captured when they are measured with one and the same task.

When independent measures of ToM and pragmatics are used the results are mixed, which appears to make sense if the two faculties are conceived of in terms of cooperation or overlap rather than identity. Langdon et al. (2002b), for example, investigated the issue in individuals with schizophrenia. They used a story-comprehension task to evaluate irony and metaphor understanding and a false belief picture-sequencing task to evaluate ToM, The latter is a non-verbal false-belief task composed by a set of picture cards representing a character who, unaware of a certain event which is instead known to the subject, acts on a false belief: goal of the task is to reorder the cards according to the logical sequence of events. The performance at the ToM task predicted the performance at irony, but not at metaphor comprehension. The authors concluded that metaphor comprehension does not involve ToM.

In a similar study, again conducted with persons with schizophrenia, Mo et al. (2008) used a story comprehension task to assess irony and metaphor understanding, in conjunction with false belief tasks to assess ToM. This time, ToM correlated with metaphor comprehension, but not with irony comprehension.

Champagne-Lavau and Stip (2010) examined the role of ToM in the comprehension of indirect requests, idiomatic (conventional) metaphorical expression, and non-idiomatic metaphors in patients with schizophrenia. Their results suggested that only indirect speech acts and idiomatic metaphors, but not non-idiomatic metaphors, are related to ToM. Mazza et al. (2008) investigated the management of Gricean conversational maxims, with similar results.

Bosco et al. (2012) found persons with schizophrenia to have both a ToM impairment and a difficulty to recognize and recover from different kinds of communicative failures, namely, in order of increasing difficulty, failure of literal meaning, speakers’ (intended) meaning, and communicative effect (e.g., the failure to induce a partner to do something). While the ability to recognize and recover from each kind of communicative failure correlated with ToM, there was no evidence that the latter was the variable that best explained the trend of difficulty, and the authors suggested that a better explanation could be provided by the increasing inferential demands underlying each task.

Finally, recently, both Bambini et al. (2016) and Parola et al. (2018) found no trace of ToM malfunction in a significant percentage of patients with schizophrenia reporting pragmatic impairment. In line with this, other studies found a ToM malfunction with no pragmatic impairment in several clinical conditions like bulimia nervosa (Laghi et al., 2014), alcoholism (Bosco et al., 2014a), and non-suicidal self-injury (Laghi et al., 2016).

Taken together, what all these data suggest is that pragmatic impairment cannot be simply reduced to a deficit of ToM; instead, a specific pragmatic ability appears to exist and function comparatively independently of ToM. This is in line with theories of human communication claiming that there exists a specific pragmatic competence, related to ToM but not identical to it (e.g., Airenti et al., 1993; Tirassa, 1999; Tirassa et al., 2006a,b; Tirassa and Bosco, 2008).

## Conclusion

While we do not think that pragmatic tasks should be used as straightforward measures of ToM, all the research we cited has valuable merit in providing significant scientific and empirical advances. We are aware that the methodological confusion we have tried to outline reflects an insufficiently explored relationship between the two theoretical constructs or faculties of pragmatics and ToM. This paper should be viewed as an attempt to highlight the magnitude of this problem in the current literature and to provide some clarifications.

An account of the relations between pragmatics and ToM should capture their similarities and differences as well as their convergences and divergences. For example, both faculties appear to require inferences; in each case, not only may or may not such inferences involve the other domain or faculty, but they are also related to other kinds of world knowledge, which in turn may or may not involve the use of language or, respectively, someone’s mental states. This may be extremely cumbersome on both theoretical and empirical grounds.

Even if these further difficulties are left aside, the relations between pragmatics and ToM are intricate and variable. We have discussed the case of the Strange Stories, taken as a valuable instance of the many tasks (and underlying perspectives) where the two faculties heavily intertwine. A case in which they can instead be kept reasonably distinct is conversational implicatures, where a listener may infer a speaker’s intended meaning from what is left unsaid, rather than from what is said. Specifically, scalar implicatures rely on quantifiers such as *some*, *all*, and so on. An example is “*On the cover of my book, some of the pictures are birds,”* whose everyday interpretation appears to imply that not all the pictures are birds. This example belongs to a set of stimuli used by Horowitz et al. (2017) to investigate the ability to deal with implicatures in TD children using sets of pictures of book covers, each featuring (a) four items of the same kind (e.g., four cats) (b) four items of another kind (e.g., dogs), and (c) two items of a new set and two items repeated from one of the other book covers (e.g., two birds and two cats). In the example presented above, no assumptions about mental states or intentions seem to be required, thus making the capacity to understand implicatures rely on inferential processes only. However, this consideration depends on the nature of the stimuli: in case the task has to do with mental states, of course, such process might involve also ToM abilities. This issue deserves further empirical investigation in order to be clarified.

Based on the available empirical literature and on the theoretical considerations briefly outlined above, we conclude that pragmatics is a faculty or construct distinct from ToM and that, while the two do partially overlap, none can be simply considered a sub-component of the other.

To avoid any confusion, it seems necessary to investigate them with distinct empirical tasks. The precise nature of the relation between pragmatics and ToM – more specifically, between different facets of pragmatics and different facets of ToM – is far from clear and further research will be needed in order to disentangle the issue.

## Author Contributions

IG, FB, and MT discussed the idea. IG and FB wrote the paper, MT revised the paper.

## Conflict of Interest Statement

The authors declare that the research was conducted in the absence of any commercial or financial relationships that could be construed as a potential conflict of interest.
